# Anisotropic dielectric susceptibility matrix of anisotropic medium

**DOI:** 10.1038/s41598-021-91435-5

**Published:** 2021-06-07

**Authors:** Wanrong Gao

**Affiliations:** Department of Optical Engineering, Nanjing University of Science and Technology, Nanjing, 200 Xiao Ling Wei, 210094 Jiangsu People’s Republic of China

**Keywords:** Polarization microscopy, Imaging and sensing

## Abstract

In this work, we introduce the concept of anisotropic dielectric susceptibility matrix of anisotropic medium for both nondepolarizing and depolarizing medium. The concept provides a new way of analyzing light scattering properties of anisotropic media illuminated by polarized light. The explicit expressions for the elements of the scattering matrix are given in terms of the elements of the Fourier transform of the anisotropic dielectric susceptibility matrix of the medium. Finally, expressions for the elements of the Jones matrix of a thin layer of a deterministic anisotropic medium and the elements of the Mueller matrix of a depolarizing medium are given. The results obtained in this work is helpful for deriving information about the correlated anisotropic structures in depolarizing media from measured Mueller matrices. The findings in this work may also well prove stimulating to researchers working on new methods for analyzing light scattering properties.

## Introduction

The knowledge about scattering of polarized light by anisotropic media has potential applications in a broad range of fields such as biomedical imaging of human tissues. Several models have then been developed that can be employed to describe the effects of an anisotropic medium on light among which the most frequently used ones are the Jones matrix formalism^1–3^ and the Mueller matrix formalism^4–6^. It is now known that the various types of anisotropic structures of media represented by polarization parameters can be derived from the measured Jones matrix and Mueller matrix by decomposing them into a product of several component matrices each of which describes a simple polarization structure when only the total averaged polarization properties of medium are concerned^7–9^. When the polarization properties at any point within a continuous anisotropic medium are desirable they can be obtained by solving a first-order linear differential Matrix equation for both nondepolarizing (deterministic) and depolarizing (random) medium^10–12^. It was found that in this case the macroscopic polarization properties can be expressed in terms of elementary polarization parameters when differential matrix is approximately a constant along the light propagation direction^13,14^.

It is a common belief that the random anisotropic medium can be described by a Mueller matrix with more than 8 degrees of freedom. To find the form of Mueller matrix of depolarizing medium, several facts about the interaction of light with medium have been exploited. Recognizing the fact that the most basic representation of an anisotropic medium is a scattering matrix (or Jones matrix) a random anisotropic medium may be modeled as fluctuations of the scattering matrix over some period of time or region of space. The appropriate description of the random media is then a statistical average of the scattering matrix over such an ensemble of the Jones matrices. With this idea it has been demonstrated by Kim et al.^15^ that the Mueller matrix of the random anisotropic medium is then proportional to an average over the ensemble of the product of the Jones matrices. One consequence of this result is that the Mueller matrix of a scattering volume $\delta V$ at point in a random anisotropic medium can be obtained by averaging Mueller–Jones matrices of the neighbor points.

Another method of describing a volume of the random anisotropic medium is to introduce a target vector^16^. The components of the target vector are defined as the complex coefficients of the expansion of the Jones matrix expanded in terms of the Pauli matrices. A 4 × 4 Hermitian target average coherency matrix is then reconstructed to describe the possible spatial correlations that exist between the fluctuations of the elements of the scattering matrix over some region within medium^14^. With the help of this concept, it is possible to find correlated anisotropic structures that are responsible for the changes of polarization properties of the incident light.

By using the coherency matrix, it has been shown that a measured Mueller matrix of a depolarizing medium can be expressed as a sum of four independent Mueller–Jones matrices each of which represents a deterministic scattering structure in a medium. The polarization properties described by the original Mueller matrix can then be calculated as the averaged polarization properties with the weighting factors given by the eigenvalues of the corresponding coherency matrix^14^. Here it is worth noting that this fact reveals again that the Mueller matrix of a depolarizing medium can be expressed in terms of an average of up to four equivalent Mueller matrices of equivalent nondepolarizing media.

This important result has been used to identify the presence of a dominating scattering matrix or average scattering matrix of the medium, to remove the experimental errors contained in the measured Mueller matrix by subtracting a Mueller matrices corresponding to the negative eigenvalues of the coherency matrix, to identity a physically realizable Mueller matrix by calculating the corresponding covariance matrix and then testing to see whether all eigenvalues are nonnegative, and to classify objects by employing the fact that the elements of a Mueller matrix can be expressed as sums of correlation coefficients of elements of scattering matrix. For example, the ratios of the eigenvalues are indicators of the anisotropy of the particles in the cloud and the eigenvectors are more indicative of any preferential alignment of the scattering particles^16^.

Notice that for continuous anisotropic medium its polarization properties may vary with position. So it is appropriate to use the differential Mueller matrix to describe its polarization properties^12^. Forms of differential Mueller matrix of deterministic and random media have been derived by several groups^17–23^. The physical meanings of the polarization parameters contained in the differential matrix have also been proposed^19–23^. In addition, coupling effects between polarization parameters have been identified and an anisotropic spatial correlation function of anisotropic random medium has been proposed to explain this phenomenon^24,25^.

Relations between properties of the scattered light and structural parameters of a medium are bases for many imaging techniques like various forms of optical tomographic microscopy^26^. For isotropic media or in the isotropic approximation, it has been shown that the scattered field in the far zone is proportional to the Fourier transform of the dielectric susceptibility of the medium for deterministic medium^26,27^ or the spectral electrical power at the detector is proportional to the Fourier transform of the spatial correlation function of the dielectric susceptibility (or refractive index)^26,28^.

However, as mentioned above, the real medium is anisotropic. It is then desirable to find a concept that characterizes light scattering by this type of media. In this work, to the best of our knowledge, for the first time the concept of anisotropic dielectric susceptibility matrix is defined of anisotropic medium for both nondepolarizing and depolarizing medium. This anisotropic dielectric susceptibility matrix is then related to the elementary polarization parameters of a nondepolarizing medium. In addition, the relationships that exist between the elements of Mueller matrix and the anisotropic spatial correlations functions of tissue refractive index is derived by using the defined anisotropic dielectric susceptibility matrix of the medium.

## Anisotropic dielectric susceptibility matrix of the medium

Now we define the dielectric susceptibility matrix of anisotropic medium. To this end, we generalize the formula describing light scattering from an isotropic statistically homogeneous medium to anisotropic random medium^26,27^. First of all, for anisotropic deterministic medium illuminated by a polarized light beam propagating in a direction specified by a unit vector $\vec{s}_{0}$, the electric field vector at $\vec{r} = r\vec{s}$ in the far zone of the scatterer can be expressed as

1 $$\left( {\begin{array}{*{20}c} {E_{sx}^{\left( \infty \right)} \left( {\vec{r},\omega } \right)} \\ {E_{sy}^{\left( \infty \right)} \left( {\vec{r},\omega } \right)} \\ \end{array} } \right) = \int_{{V\left( {\vec{r}^{\prime}} \right)}} {\left( {\begin{array}{*{20}c} {\eta_{11} \left( {\vec{r}^{\prime},\omega } \right)} & {\eta_{12} \left( {\vec{r}^{\prime},\omega } \right)} \\ {\eta_{21} \left( {\vec{r}^{\prime},\omega } \right)} & {\eta_{22} \left( {\vec{r}^{\prime},\omega } \right)} \\ \end{array} } \right)} \exp \left[ { - j\vec{K} \cdot \left( {\vec{r} - \vec{r}^{\prime}} \right)} \right]d^{3} r^{\prime}\left( {\begin{array}{*{20}c} {E_{x0} } \\ {E_{y0} } \\ \end{array} } \right)k^{2} a\left( \omega \right)\frac{{e^{{jk\vec{s}_{0} \cdot \vec{r}}} }}{r},$$

where $k$ is the wavenumber in free space, $a\left( \omega \right)$ is a function of frequency, c denotes the speed of light in vacuo, ω is the angular frequency, $\vec{r}^{\prime}$ is the position vector in the medium, $r$ denotes the magnitude of the vector $\vec{r} = r\vec{s}$ ($\vec{s}^{2} = 1$) from the reference point in the medium to the observation point, $\vec{s}$ denotes a unit vector along the observation direction, $\vec{K} = k\left( {\vec{s} - \vec{s}_{0} } \right)$ is the scattering vector, $$\left( {\begin{array}{*{20}c} {E_{x0} } & {E_{y0} } \\ \end{array} } \right)^{T} a\left( \omega \right)e^{{jk\vec{s}_{0} }}$$ represents the incident electric field vector, where the superscript T denotes the transpose, and $V$ denotes the illuminated volume.

In Eq. (1) we have defined the anisotropic dielectric susceptibility matrix ${\mathbf{H}}$ of the medium the elements of which are defined by

2 $$\eta_{ij} = {{\left[ {\tilde{n}_{i} \tilde{n}_{j}^{ * } - 1} \right]} \mathord{\left/ {\vphantom {{\left[ {\tilde{n}_{i} \tilde{n}_{j}^{ * } - 1} \right]} {4\pi }}} \right. \kern-\nulldelimiterspace} {4\pi }},$$

where $\tilde{n}_{i} \left( {i = 1,2} \right)$ denotes the complex refractive index along the two coordinate axes, and * denotes the complex conjugate. Here for brevity we have omitted the variables in the functions $\eta_{ij}$ and $\tilde{n}_{i}$.

Equation (1) provides a new base for analyzing light scattering properties of anisotropic media. Note that when coordinate axes are along the two principle axes of the medium (for example, $i,j = x,y$) the anisotropic dielectric susceptibility matrix is of the diagonal form. It is worth noting that for the isotropic media, Eq. (1) reduces to the familiar result of the scalar isotropic scattering theory^26,27^.

Equation (1) shows that for anisotropic deterministic medium the electric field vector at $\vec{r} = r\vec{s}$ in the far zone of the scatterer is determined by the Fourier transform of the anisotropic dielectric susceptibility matrix of the medium. Note that this result is based the first Born approximation to the scattered light and is a generalization of the light scattering properties of the isotropic media^26,27^.

Equation (1) can formally be rewritten as

3 $$\left( {\begin{array}{*{20}c} {E_{sx}^{\left( \infty \right)} \left( {\vec{r},\omega } \right)} \\ {E_{sy}^{\left( \infty \right)} \left( {\vec{r},\omega } \right)} \\ \end{array} } \right) = \left( {\begin{array}{*{20}c} {J_{11} } & {J_{12} } \\ {J_{21} } & {J_{22} } \\ \end{array} } \right)\left( {\begin{array}{*{20}c} {E_{x0} } \\ {E_{y0} } \\ \end{array} } \right)a\left( \omega \right)e^{{jk\vec{s}_{0} }} .$$

A comparison of Eq. (3) with Eq. (1) shows that the Jones matrix (or scattering matrix) of the medium is proportional to the Fourier transform of the anisotropic dielectric susceptibility matrix of the medium which is defined by Eq. (2) in terms of the refractive index along the two coordinate axes:

4 $$\left( {\begin{array}{*{20}l} {J_{11} } & {J_{12} } \\ {J_{21} } & {J_{22} } \\ \end{array} } \right) = \int_{{V\left( {\vec{r}^{\prime}} \right)}} {\left( {\begin{array}{*{20}c} {\eta_{11} \left( {\vec{r}^{\prime},\omega } \right)} & {\eta_{12} \left( {\vec{r}^{\prime},\omega } \right)} \\ {\eta_{21} \left( {\vec{r}^{\prime},\omega } \right)} & {\eta_{22} \left( {\vec{r}^{\prime},\omega } \right)} \\ \end{array} } \right)} \exp \left[ { - j\vec{K} \cdot \left( {\vec{r} - \vec{r}^{\prime}} \right)} \right]d^{3} r^{\prime}\frac{1}{r}\left( {\frac{\omega }{c}} \right)^{2} .$$

Equation (4) is the key result in this work. It provides explicit expressions for the elements of the scattering matrix of the anisotropic medium in the form of Fourier transform of the elements of the anisotropic dielectric susceptibility matrix.

Note that for deterministic medium the Jones matrix of the medium of the thickness $\Delta z$ can be expressed explicitly in terms of the elementary polarization parameters^11^:

5 $${\mathbf{J}} = \exp \left( {T\Delta z} \right)\left( {\begin{array}{*{20}l} {\cosh \left( {Q\Delta z} \right) + \frac{1}{2}\left( {n_{1} - n_{2} } \right)\frac{{\sinh \left( {Q\Delta z} \right)}}{Q}} & {n_{4} \frac{{\sinh \left( {Q\Delta z} \right)}}{Q}} \\ {n_{3} \frac{{\sinh \left( {Q\Delta z} \right)}}{Q}} & {\cosh \left( {Q\Delta z} \right) - \frac{1}{2}\left( {n_{1} - n_{2} } \right)\frac{{\sinh \left( {Q\Delta z} \right)}}{Q}} \\ \end{array} } \right),$$

where

6 $$T = \frac{\alpha }{2} + i\frac{\varphi }{2},\,Q^{2} = \left( {\frac{\beta }{2} + i\frac{\eta }{2}} \right)^{2} + \left( {\frac{\gamma }{2} - i\frac{\nu }{2}} \right)^{2} - \left( { - \frac{\mu }{2} + i\frac{\delta }{2}} \right)^{2} ,\;n_{1} = \frac{\alpha }{2} + \frac{\beta }{2} + i\frac{\varphi }{2} + i\frac{\eta }{2},$$

7 $$n_{2} = \frac{\alpha }{2} - \frac{\beta }{2} + i\frac{\varphi }{2} - i\frac{\eta }{2},\;n_{3} = - \frac{\mu }{2} + \frac{\gamma }{2} + i\frac{\delta }{2} - i\frac{\nu }{2},\;n_{4} = \frac{\mu }{2} + \frac{\gamma }{2} - i\frac{\delta }{2} - i\frac{\nu }{2},\;n_{1} - n_{2} = \beta + i\eta ,$$

where $\varphi$ is the phase retardation per unit thickness. Note that Eq. (5) is valid when the differential Jones matrix can be regarded as a constant over the illuminated volume of the medium.

Note that Eqs. (4) and (5) are expressions of the Jones matrix for the same medium and their right-hand sides must be equal. Thus a combination of Eq. (4) with Eq. (5) allows us to clearly relate the anisotropic dielectric susceptibility matrix of the medium to elementary polarization parameters. This fact may be used to model the sources of the elementary polarization parameters in terms of the anisotropic refractive indices (see Eq. (2)). For example, to determine the contributions of the complex refractive index to the values of the polarization parameters. In addition, it also allows us to determine the polarization properties of medium from the measured scattering matrix of the thin layer of medium.

Equation (5) reveals that the anisotropic refractive index $\tilde{n}_{i} \left( {i = 1,2} \right)$ used in Eq. (2) for defining the anisotropic dielectric susceptibility matrix should be complex numbers that contain all the polarization properties of the medium such as the circular birefringence and dichroism.

For depolarizing (or random) medium Eq. (2) should be

8 $$\eta_{ij} = {{\left[ {\left\langle {\tilde{n}_{i} \tilde{n}_{j}^{ * } } \right\rangle - 1} \right]} \mathord{\left/ {\vphantom {{\left[ {\left\langle {\tilde{n}_{i} \tilde{n}_{j}^{ * } } \right\rangle - 1} \right]} {4\pi }}} \right. \kern-\nulldelimiterspace} {4\pi }},$$

where $\left\langle \cdots \right\rangle$ denotes the ensemble average. In this case, $\left\langle {\tilde{n}_{i} \tilde{n}_{j}^{ * } } \right\rangle$ is the spatial correlation function of the anisotropic refractive index $\tilde{n}_{i} \left( {i = 1,2} \right)$. Equation (4) can be regarded as a realization of the ensemble representing a medium exhibiting spatial, or temporal fluctuations over the area illuminated by the incident light with some probability. Equation (4) then reveals that the realization of the Jones matrix of the depolarizing medium is proportional to the Fourier transform of the realization of the anisotropic dielectric susceptibility matrix of the medium.

## Relating elements of macroscopic Mueller matrix to correlations of elements of Jones matrices

Now we consider an application of relation (4) to obtain expressions for macroscopic Mueller matrix of a thin layer of medium beneath the sample surface. It has been demonstrated that when an ensemble of Jones matrices is used to represent a random linear medium, the effects of an ensemble of Jones matrices are equivalent to the Mueller matrix^15^. The elements $m_{\mu \nu } \left( {\Delta z} \right)$ of the Mueller matrix ${\mathbf{M}}\left( {\Delta z} \right)$ can be expressed as^15^:

9 $$m_{\mu \nu } \left( {\Delta z} \right) = \frac{1}{2}Tr\left[ {\sigma^{\left( \mu \right)} J\sigma^{\left( \nu \right)} J^{\dag } } \right] = \frac{1}{2}\sum\limits_{n = 1}^{2} {\sum\limits_{p = 1}^{2} {\sum\limits_{q = 1}^{2} {\sum\limits_{m = 1}^{2} {\left\langle {J_{np}^{\left( e \right)} J_{qm}^{\left( e \right)\dag } } \right\rangle_{e} \sigma_{mn}^{\left( \mu \right)} \sigma_{pq}^{\left( \nu \right)} } } } } ,\quad \left( {\mu ,\nu = 0,1,2,3} \right),$$

where $\left\langle {} \right\rangle_{e}$ denotes average over the ensemble, † denotes the Hermitian adjoint, $Tr\left[ {} \right]$ represents the trace of the matrix, $J^{\left( e \right)}$ is a typical element of the ensemble of a 2 × 2 Jones matrix of the thin slab, $\sigma^{\left( \mu \right)}$ ($\mu = 0,1,2,3$) are the four linearly independent 2 × 2 Pauli matrices

10 $$\sigma^{\left( 0 \right)} = \left( {\begin{array}{*{20}c} 1 & 0 \\ 0 & 1 \\ \end{array} } \right),\;\sigma^{\left( 1 \right)} = \left( {\begin{array}{*{20}c} 1 & 0 \\ 0 & { - 1} \\ \end{array} } \right),\;\sigma^{\left( 2 \right)} = \left( {\begin{array}{*{20}c} 0 & 1 \\ 1 & 0 \\ \end{array} } \right),\;\sigma^{\left( 3 \right)} = \left( {\begin{array}{*{20}c} 0 & { - i} \\ i & 0 \\ \end{array} } \right).$$

Substituting Eq. (10) into Eq. (9) gives us

11 $$m_{00} = \frac{1}{2}\left( {\left\langle {J_{11} J_{11}^{ * } } \right\rangle_{e} + \left\langle {J_{12} J_{12}^{ * } } \right\rangle_{e} + \left\langle {J_{21} J_{21}^{ * } } \right\rangle_{e} + \left\langle {J_{22} J_{22}^{ * } } \right\rangle_{e} } \right),$$

12 $$m_{01} = \frac{1}{2}\left( {\left\langle {J_{11} J_{11}^{ * } } \right\rangle_{e} - \left\langle {J_{12} J_{12}^{ * } } \right\rangle_{e} + \left\langle {J_{21} J_{21}^{ * } } \right\rangle_{e} - \left\langle {J_{22} J_{22}^{ * } } \right\rangle_{e} } \right),$$

13 $$m_{02} = \frac{1}{2}\left( {\left\langle {J_{11} J_{12}^{ * } } \right\rangle_{e} + \left\langle {J_{12} J_{11}^{ * } } \right\rangle_{e} + \left\langle {J_{21} J_{22}^{ * } } \right\rangle_{e} + \left\langle {J_{22} J_{21}^{ * } } \right\rangle_{e} } \right),$$

14 $$m_{03} = \frac{1}{2}\left( { - i\left\langle {J_{11} J_{12}^{ * } } \right\rangle_{e} + i\left\langle {J_{12} J_{11}^{ * } } \right\rangle_{e} - i\left\langle {J_{21} J_{22}^{ * } } \right\rangle_{e} + i\left\langle {J_{22} J_{21}^{ * } } \right\rangle_{e} } \right),$$

15 $$m_{10} = \frac{1}{2}\left( {\left\langle {J_{11} J_{11}^{ * } } \right\rangle_{e} + \left\langle {J_{12} J_{12}^{ * } } \right\rangle_{e} - \left\langle {J_{21} J_{21}^{ * } } \right\rangle_{e} - \left\langle {J_{22} J_{22}^{ * } } \right\rangle_{e} } \right),$$

16 $$m_{11} = \frac{1}{2}\left( {\left\langle {J_{11} J_{11}^{ * } } \right\rangle_{e} - \left\langle {J_{12} J_{12}^{ * } } \right\rangle_{e} - \left\langle {J_{21} J_{21}^{ * } } \right\rangle_{e} + \left\langle {J_{22} J_{22}^{ * } } \right\rangle_{e} } \right),$$

17 $$m_{12} = \frac{1}{2}\left( {\left\langle {J_{11} J_{12}^{ * } } \right\rangle_{e} + \left\langle {J_{12} J_{11}^{ * } } \right\rangle_{e} - \left\langle {J_{21} J_{22}^{ * } } \right\rangle_{e} - \left\langle {J_{22} J_{21}^{ * } } \right\rangle_{e} } \right),$$

18 $$m_{13} = \frac{1}{2}\left( { - i\left\langle {J_{11} J_{12}^{ * } } \right\rangle_{e} + i\left\langle {J_{12} J_{11}^{ * } } \right\rangle_{e} + i\left\langle {J_{21} J_{22}^{ * } } \right\rangle_{e} - i\left\langle {J_{22} J_{21}^{ * } } \right\rangle_{e} } \right),$$

19 $$m_{20} = \frac{1}{2}\left( {\left\langle {J_{21} J_{11}^{ * } } \right\rangle_{e} + \left\langle {J_{22} J_{12}^{ * } } \right\rangle_{e} + \left\langle {J_{11} J_{21}^{ * } } \right\rangle_{e} + \left\langle {J_{12} J_{22}^{ * } } \right\rangle_{e} } \right),$$

20 $$m_{21} = \frac{1}{2}\left( {\left\langle {J_{21} J_{11}^{ * } } \right\rangle_{e} - \left\langle {J_{22} J_{12}^{ * } } \right\rangle_{e} + \left\langle {J_{11} J_{21}^{ * } } \right\rangle_{e} - \left\langle {J_{12} J_{22}^{ * } } \right\rangle_{e} } \right),$$

21 $$m_{22} = \frac{1}{2}\left( {\left\langle {J_{21} J_{12}^{ * } } \right\rangle_{e} + \left\langle {J_{22} J_{11}^{ * } } \right\rangle_{e} + \left\langle {J_{11} J_{22}^{ * } } \right\rangle_{e} + \left\langle {J_{12} J_{21}^{ * } } \right\rangle_{e} } \right),$$

22 $$m_{23} = \frac{1}{2}\left( { - i\left\langle {J_{21} J_{12}^{ * } } \right\rangle_{e} + i\left\langle {J_{22} J_{11}^{ * } } \right\rangle_{e} - i\left\langle {J_{11} J_{22}^{ * } } \right\rangle_{e} + i\left\langle {J_{12} J_{21}^{ * } } \right\rangle_{e} } \right),$$

23 $$m_{30} = \frac{1}{2}\left( { - i\left\langle {J_{21} J_{11}^{ * } } \right\rangle_{e} - i\left\langle {J_{22} J_{12}^{ * } } \right\rangle_{e} + i\left\langle {J_{11} J_{21}^{ * } } \right\rangle_{e} + i\left\langle {J_{12} J_{22}^{ * } } \right\rangle_{e} } \right),$$

24 $$m_{31} = \frac{1}{2}\left( { - i\left\langle {J_{21} J_{11}^{ * } } \right\rangle_{e} + i\left\langle {J_{22} J_{12}^{ * } } \right\rangle_{e} + i\left\langle {J_{11} J_{21}^{ * } } \right\rangle_{e} - i\left\langle {J_{12} J_{22}^{ * } } \right\rangle_{e} } \right),$$

25 $$m_{32} = \frac{1}{2}\left( { - i\left\langle {J_{21} J_{12}^{ * } } \right\rangle_{e} - i\left\langle {J_{22} J_{11}^{ * } } \right\rangle_{e} + i\left\langle {J_{11} J_{22}^{ * } } \right\rangle_{e} + i\left\langle {J_{12} J_{21}^{ * } } \right\rangle_{e} } \right),$$

26 $$m_{33} = \frac{1}{2}\left( { - \left\langle {J_{21} J_{12}^{ * } } \right\rangle_{e} + \left\langle {J_{22} J_{11}^{ * } } \right\rangle_{e} + \left\langle {J_{11} J_{22}^{ * } } \right\rangle_{e} - \left\langle {J_{12} J_{21}^{ * } } \right\rangle_{e} } \right).$$

Noting that Eq. (9) shows that the elements of the macroscopic Mueller matrix of the thin layer are determined by an ensemble average of the product of two Jones matrices of the same medium. Specifically, as can be seen from Eqs. (11)–(26), the elements of the macroscopic Mueller matrix of a thin slice are sums of combinations of the products of two elements of the Jones matrix.

Assuming that

27 $$\tilde{n}_{i} = \left( {\left\langle {n_{i} } \right\rangle + \delta n_{i} } \right) - j\left( {\left\langle {\alpha_{i} } \right\rangle + \delta \alpha_{i} } \right)\quad \left( {i = 1,2} \right),$$

where $n_{i} \left( {i = x,y} \right)$ are the principal indices of refraction and $\alpha_{i} \left( {i = x,y} \right)$ are the principal extinction coefficients, $\left\langle {n_{i} } \right\rangle$ and $\left\langle {n_{i} } \right\rangle$ is the mean values of $n_{i}$ and $\alpha_{i}$, respectively; $\delta n_{i} \left( {i = 1,2} \right)$ are the varying parts of the refractive indices with $\left\langle {\delta n_{i} } \right\rangle = 0\left( {i = 1,2} \right)$, and $\delta \alpha_{i} \left( {i = 1,2} \right)$ are the variations of the principal absorption coefficients with $\left\langle {\delta \alpha_{i} } \right\rangle = 0\left( {i = x,y} \right)$, respectively. Note that, in general, the parameters $n_{i} \left( {i = 1,2} \right)$ and $\alpha_{i} \left( {i = 1,2} \right)$ are functions of the position $\vec{r}$ within the medium.

On substituting Eqs. (2) and (4) into Eqs. (11)–(26) and assuming that the fluctuations of the complex anisotropic refractive indices $\tilde{n}_{i} \left( {i = 1,2} \right)$ are very small compared with their mean values, the terms can be neglected that are expressed as products in terms of $\delta n_{i} \left( {i = 1,2} \right)$ and $\delta \alpha_{i} \left( {i = 1,2} \right)$ with the exponents of $\delta n_{i} \left( {i = 1,2} \right)$ and $\delta \alpha_{i} \left( {i = 1,2} \right)$ total being larger than 4. After very long and straightforward calculations we then obtain a correlation matrix ${\mathbf{C}}_{S}$ of the medium generated by its anisotropic dielectric susceptibility matrix (see Eq. (1)) each element of which is a sum of the correlations between the fluctuations of the refractive indices and absorption coefficients of the complex refractive indices $\tilde{n}_{i} \left( {i = 1,2} \right)$.

## Expressions for differential Mueller matrix of a thin layer of medium

Now we consider the expressions for differential Mueller matrix ${\mathbf{m}}$ of medium. For a thin slab of medium of thickness $\Delta z \to 0$, its differential Mueller matrix ${\mathbf{m}}$ can be approximated as a constant. In this case, its macroscopic Mueller matrix ${\mathbf{M}}\left( {\Delta z} \right)$ can be expressed as

28 $${\mathbf{M}}\left( {\Delta z} \right){ = }{\mathbf{I}}{ + }{\mathbf{m}}\Delta z,$$

where ${\mathbf{I}}$ is the identity matrix and ${\mathbf{m}}$ can be expressed as a sum of two terms^19^

29 $${\mathbf{m}} = {\mathbf{m}}_{nondep} + {\mathbf{m}}_{dep} ,$$

where ${\mathbf{m}}_{nondep}$ contains the elementary polarization properties (per unit length) of the medium, and the elements of matrix ${\mathbf{m}}_{dep}$ are the depolarizing properties of the medium. The form of ${\mathbf{m}}_{nondep}$ was derived by Azzam and is given by^12^

30 $${\mathbf{m}}_{nondep} = \left| {\begin{array}{*{20}c} \alpha & \beta & \gamma & \delta \\ \beta & \alpha & \mu & \nu \\ \gamma & { - \mu } & \alpha & \eta \\ \delta & { - \nu } & { - \eta } & \alpha \\ \end{array} } \right|,$$

where α is the isotropic absorption, β is the linear dichroism along the x–y laboratory axes per unit distance, γ is the linear dichroism along the ± 45° axes per unit distance, and δ is the circular dichroism, η is linear birefringence along the x–y laboratory axes per unit distance, ν is the linear birefringence along the ± 45° axes per unit distance, and μ is the circular birefringence. Several forms of ${\mathbf{m}}_{dep}$ have been derived based on different facts^20–25^.

Equation (28) can then be expressed as

31 $${\mathbf{M}}\left( {\Delta z} \right){ = }{\mathbf{I}}{ + }{\mathbf{m}}\Delta z = \Xi {\tilde{\mathbf{C}}}_{S} ,$$

where $\Xi$ is a coefficient and ${\tilde{\mathbf{C}}}_{S}$ is the Fourier transform of a correlation matrix ${\mathbf{C}}_{S}$ defined by Eq. (4). From Eq. (31) we also have

32 $${\mathbf{m}}\Delta z = \Xi {\tilde{\mathbf{C}}} - {\mathbf{I}}.$$

Equation (32) shows that the differential polarization parameters are directly related to the spatial Fourier transform of the anisotropic spatial correlation function of the refractive index of the medium.

Note that our result is a generalization of the scalar solution of the Maxwell equation of light scattering by the isotropic media^27^. In our analysis, by introducing the anisotropic dielectric susceptibility matrix of the medium, it is possible to directly relate the differential polarization parameters to the anisotropic structures of media.

## An example

As an example of the possible applications of Eq. (31), we now try to calculate the elements of the macroscopic Mueller matrix of a thin slice of medium and compare them with the expressions for the elements of the depolarizing part of the differential Mueller matrix of random continuous anisotropic medium derived by Devlaminck^21,22^.

Starting from the differential equation of random differential Mueller matrix and assuming that the random fluctuations of the differential polarization parameters are with a short correlation distance and are with Gaussian white noise-like, it has been shown that the z-independent depolarizing differential Mueller matrix can be expressed as^22^:

33 $${\mathbf{m}} = {\mathbf{m}}_{nondep} + {\mathbf{m}}_{dep} ,$$

34 $${\mathbf{m}}_{dep} = \left( {\begin{array}{*{20}l} {\left\langle {\sigma_{4}^{2} } \right\rangle + \left\langle {\sigma_{5}^{2} } \right\rangle + \left\langle {\sigma_{6}^{2} } \right\rangle } & { - \frac{1}{2}\left( {\left\langle {\sigma_{2} \sigma_{6} } \right\rangle - \left\langle {\sigma_{3} \sigma_{5} } \right\rangle } \right)} & { - \frac{1}{2}\left( {\left\langle {\sigma_{3} \sigma_{4} } \right\rangle - \left\langle {\sigma_{1} \sigma_{6} } \right\rangle } \right)} & { - \frac{1}{2}\left( {\left\langle {\sigma_{1} \sigma_{5} } \right\rangle - \left\langle {\sigma_{2} \sigma_{4} } \right\rangle } \right)} \\ {\frac{1}{2}\left( {\left\langle {\sigma_{2} \sigma_{6} } \right\rangle - \left\langle {\sigma_{3} \sigma_{5} } \right\rangle } \right)} & {\left\langle {\sigma_{4}^{2} } \right\rangle - \left\langle {\sigma_{2}^{2} } \right\rangle - \left\langle {\sigma_{3}^{2} } \right\rangle } & {\frac{1}{2}\left( {\left\langle {\sigma_{1} \sigma_{2} } \right\rangle - \left\langle {\sigma_{4} \sigma_{5} } \right\rangle } \right)} & {\frac{1}{2}\left( {\left\langle {\sigma_{1} \sigma_{3} } \right\rangle - \left\langle {\sigma_{4} \sigma_{6} } \right\rangle } \right)} \\ {\frac{1}{2}\left( {\left\langle {\sigma_{3} \sigma_{4} } \right\rangle - \left\langle {\sigma_{1} \sigma_{6} } \right\rangle } \right)} & {\frac{1}{2}\left( {\left\langle {\sigma_{1} \sigma_{2} } \right\rangle - \left\langle {\sigma_{4} \sigma_{5} } \right\rangle } \right)} & {\left\langle {\sigma_{5}^{2} } \right\rangle - \left\langle {\sigma_{1}^{2} } \right\rangle + \left\langle {\sigma_{3}^{2} } \right\rangle } & {\frac{1}{2}\left( {\left\langle {\sigma_{2} \sigma_{3} } \right\rangle - \left\langle {\sigma_{5} \sigma_{6} } \right\rangle } \right)} \\ {\frac{1}{2}\left( {\left\langle {\sigma_{1} \sigma_{5} } \right\rangle - \left\langle {\sigma_{2} \sigma_{4} } \right\rangle } \right)} & {\frac{1}{2}\left( {\left\langle {\sigma_{1} \sigma_{3} } \right\rangle - \left\langle {\sigma_{4} \sigma_{6} } \right\rangle } \right)} & {\frac{1}{2}\left( {\left\langle {\sigma_{2} \sigma_{3} } \right\rangle - \left\langle {\sigma_{5} \sigma_{6} } \right\rangle } \right)} & {\left\langle {\sigma_{6}^{2} } \right\rangle - \left\langle {\sigma_{1}^{2} } \right\rangle - \left\langle {\sigma_{2}^{2} } \right\rangle } \\ \end{array} } \right),$$

35 $${\mathbf{m}}_{nondep} = \left( {\begin{array}{*{20}l} {\mu_{0} + \left\langle {\sigma_{0}^{2} } \right\rangle } & {\mu_{4} + 2\left\langle {\sigma_{0} \sigma_{4} } \right\rangle } & {\mu_{5} + 2\left\langle {\sigma_{0} \sigma_{5} } \right\rangle } & {\mu_{6} + 2\left\langle {\sigma_{0} \sigma_{6} } \right\rangle } \\ {\mu_{4} + 2\left\langle {\sigma_{0} \sigma_{4} } \right\rangle } & {\mu_{0} + \left\langle {\sigma_{0}^{2} } \right\rangle } & { - \mu_{3} - 2\left\langle {\sigma_{0} \sigma_{3} } \right\rangle } & {\mu_{2} + 2\left\langle {\sigma_{0} \sigma_{2} } \right\rangle } \\ {\mu_{5} + 2\left\langle {\sigma_{0} \sigma_{5} } \right\rangle } & {\mu_{3} + 2\left\langle {\sigma_{0} \sigma_{3} } \right\rangle } & {\mu_{0} + \left\langle {\sigma_{0}^{2} } \right\rangle } & { - \mu_{1} - 2\left\langle {\sigma_{0} \sigma_{1} } \right\rangle } \\ {\mu_{6} + 2\left\langle {\sigma_{0} \sigma_{6} } \right\rangle } & { - \mu_{2} - 2\left\langle {\sigma_{0} \sigma_{2} } \right\rangle } & {\mu_{1} + 2\left\langle {\sigma_{0} \sigma_{1} } \right\rangle } & {\mu_{0} + \left\langle {\sigma_{0}^{2} } \right\rangle } \\ \end{array} } \right),$$

where $\mu_{i}$ ($i = 0,1, \ldots ,6$) are mean values of the elementary polarization properties of $p_{i} \left( z \right)$, $\sigma_{ij}$($i,j = 0,1, \cdots ,6$) are entries of the covariance matrix of the centered Gaussian white noses $p_{i} \left( z \right)$, $\sigma_{i}^{2} = \sigma_{ii}$ is the variance of the process^22^:

36 $$\left\langle {p_{i} \left( z \right)} \right\rangle = \mu_{i} ,\;\left\langle {\left( {p_{i} \left( {z_{2} } \right) - \mu_{i} } \right)\left( {p_{j} \left( {z_{1} } \right) - \mu_{j} } \right)} \right\rangle = \sigma_{ij} \delta \left( {z_{2} - z_{1} } \right),\;\left( {i = 0,1, \ldots ,6} \right).$$

Note that for simplicity, the elementary polarization parameters (α, η, ν, μ, β, γ, δ) have been denoted by $\mu_{i}$ ($i = 0,1, \ldots ,6$). Equations (34) and (35) show that the correlations between the fluctuations of the elementary polarization properties contribute to both the mean values of the nondepolarizing properties and the depolarizing polarization properties (or the uncertainties of polarization properties). They also show that only for deterministic media or there is no spatial correlation between the fluctuations of the elementary optical properties the mean values of the nondepolarizing properties take the values of the elementary parameters.

It is worth noting that the spatial correlations come from the correlations of the processes associated with complementary optical properties that contribute to the depolarizing properties. For example, the uncertainty of the linear birefringence along the x–y laboratory axes arises from the spatial correlations between the fluctuations of birefringence along the 45° ($\left\langle {\sigma_{2} \sigma_{3} } \right\rangle$) and circular directions as well as the spatial correlations between the fluctuations of attenuation along the 45° and circular directions ($\left\langle {\sigma_{5} \sigma_{6} } \right\rangle$), respectively.

Now we show that for a thin layer of the medium Eqs. (34) and (35) can be derived from our results. As an example, we only consider the case in which the absorbance of the medium is small and can be neglected. In this case, Eq. (5) then simplifies to

37 $${\mathbf{J}} = \exp \left( {i\frac{1}{2}\varphi \Delta z} \right)\left( {\begin{array}{*{20}c} {\cos \left( {\Gamma \Delta z} \right) + \frac{1}{2}\left( {ip_{1} } \right)\frac{{\sin \left( {\Gamma \Delta z} \right)}}{\Gamma }} & {\left( {\frac{{p_{3} }}{2} - i\frac{{p_{2} }}{2}} \right)\frac{{\sin \left( {\Gamma \Delta z} \right)}}{\Gamma }} \\ {\left( { - \frac{{p_{3} }}{2} - i\frac{{p_{2} }}{2}} \right)\frac{{\sin \left( {\Gamma \Delta z} \right)}}{\Gamma }} & {\cos \left( {\Gamma \Delta z} \right) - \frac{1}{2}\left( {ip_{1} } \right)\frac{{\sin \left( {\Gamma \Delta z} \right)}}{\Gamma }} \\ \end{array} } \right),$$

where

38 $$\Gamma^{2} = \frac{1}{4}\left( {p_{1}^{2} + p_{2}^{2} + p_{3}^{2} } \right).$$

By using Eqs. (11–26) and (36) we can obtain the expressions for the elements of the macroscopic Mueller matrix ${\mathbf{M}}\left( {\Delta z} \right)$ of the thin layer. For example, $m_{23}$ can be expressed as

39 $$m_{23} = \mu_{1} \cos \left( {\Gamma \Delta z} \right)\frac{{\sin \left( {\Gamma \Delta z} \right)}}{\Gamma } - \frac{1}{2}\left( {\mu_{2} \mu_{3} + \left\langle {\sigma_{2} \sigma_{3} } \right\rangle } \right).$$

A comparison of Eq. (39) with the corresponding elements of the differential Mueller matrices ${\mathbf{m}}_{nondep}$ and ${\mathbf{m}}_{dep}$ in Eqs. (34) and (35) reveals that for a thin slice of the medium, like its differential Mueller matrix, the elements of the macroscopic Mueller matrix can also be expressed as a sum of terms being linear to their mean values and the terms being linear to the correlations of two fluctuations.

This result suggests that a combination of relations Eqs. (4), (5), (9), (11)–(26) and (31) allows us to predict the values of the elements of macroscopic Mueller matrix ${\mathbf{M}}\left( {\Delta z} \right)$ of the thin layer with given polarization properties. They can also be employed to find polarization phenomena from measured Mueller matrix which are significant in the medium under test.

## Discussions

The main result of this work is the definition of the anisotropic dielectric susceptibility matrix ${\mathbf{H}}$ of the medium (Eqs. (1), (4)), and thus generalize the well-known isotropic scattering formula (Eqs. (24), (25) in chapter 13 in ref.^27^) to light scattering by anisotropic media. Many possible applications are expected. First, this concept makes it possible to relate the electric vector of the light scattered by anisotropic media to their anisotropic dielectric susceptibility. Second, it can be used to calculate the scattering matrix for nondepolarizing media. For a depolarizing medium it can be employed to find the Mueller matrices of the thin layer of the media. Third, a combination Eqs. (4), (7) and (11)–(26) allows us to find the correlated anisotropic structures from the measured macroscopic Mueller matrices. Finally, the various form of Fourier transform relations between the Jones matrix of and the anisotropic dielectric susceptibility matrix of the medium allow us to simulate the polarization properties of a modeled tissue with given values of polarization parameters.

## Conclusions

In this work, a theoretical model is proposed that can relate the optically anisotropic structures of a generally depolarizing medium to its Mueller matrix. We generalize the formula that describes light scattering from an isotropic statistically homogeneous tissues to anisotropic random medium by defining an anisotropic spatial correlation of the anisotropic complex refractive index and the anisotropic dielectric susceptibility matrix of the medium. The fact that the Mueller matrix of a medium contains all the polarimetric information that is possible to get from the medium suggests that our theory can be used to derive the model of medium with required polarization properties, to simulate the light scattering of incident polarized light by a thin slab of the given anisotropic media at a depth beneath the surface or at the sample surface, and to interpret the measured Mueller matrix by relating the measured polarization parameters to the three-dimensional anisotropic structures of the medium.
